# Inferring the annual migration patterns of fall armyworm (Lepidoptera: Noctuidae) in the United States from mitochondrial haplotypes

**DOI:** 10.1002/ece3.268

**Published:** 2012-07

**Authors:** Rodney N Nagoshi, Robert L Meagher, Mirian Hay-Roe

**Affiliations:** Center for Medical, Agricultural and Veterinary EntomologyUSDA-ARS, Gainesville, Florida 32608

**Keywords:** Migration, molecular haplotypes, *Spodoptera frugiperda*

## Abstract

*Spodoptera frugiperda* (J. E. Smith) or fall armyworm is an important agricultural pest of a number of crops in the western hemisphere. In the United States, infestations in corn acreages extend from the Mexican to the Canadian border. Because fall armyworm does not survive prolonged freezing, the infestations annually affecting most of North America are migrants from southern Texas and Florida, where winter temperatures are mild and host plants are available. A haplotype method was developed that can distinguish between these two geographically distant overwintering populations, with the potential to delineate the associated migratory pathways. Several years of collections from major corn-producing areas in the southern, central, and eastern United States were used to map the geographical distribution of the fall armyworm haplotypes. From these haplotype profiles, it was possible to develop the most detailed description yet of the annual northward movements of fall armyworm. The consistency of these results with past studies and the implications on our understanding of fall armyworm biology are discussed. A better understanding of fall armyworm populations and their movement is critical for the development of strategies to predict infestation levels and eventually control this pest in the United States.

## Introduction

**Figure 1:**
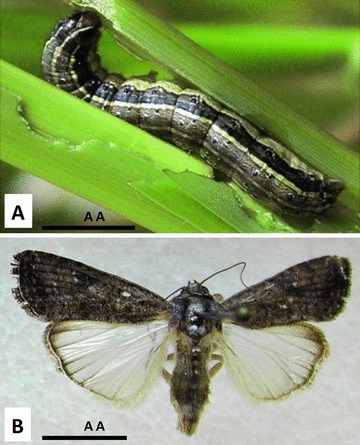
Fall armyworm (*Spodoptera frugiperda*) larva and adult. (A) Late instar larva feeding on corn (Courtesy: C. Stuhl, USDA-ARS). (B) Adult male from pheromone trap (Courtesy: M. Hay-Roe, USDA-ARS).

Annual long-distance migration is a behavioral adaptation used by many Lepidoptera species to extend their geo-graphical range into areas that cannot support permanent populations. A well-known example in North America is the fall armyworm, *Spodoptera frugiperda* (J. E. Smith) (Fig. 1). This member of the Noctuidae family does not survive severe winters, yet infestations annually occur throughout the central and eastern United States, extending as far north as Canada. The infesting populations are migrants that overwinter in southern Florida and southern Texas–Mexico (Luginbill 1928). This recurring pattern of large-scale population movements from defined source locations make fall armyworm a potentially powerful model system to study factors affecting the long-range movements of flying insects (Muller 1985; Wolf et al. 1990; Johnson 1995).

Additional interest in fall armyworm migration behavior stems from the recent discovery in Puerto Rico populations of a naturally occurring resistance to a Bacillus thurigiensis protein used in several commercially available genetically modified corn lines (Storer et al. 2010). Characterization of the fall armyworm migratory pathways could identify regions at risk for increased monitoring and preemptive control measures.

Early descriptions of fall armyworm migration were inferences based upon the timing of first appearances in progressively northern sites (Luginbill 1928; Pair et al. 1986). These approximations were complemented by extrapolations of likely flight patterns based on synoptic meteorological conditions and seasonal wind patterns (Rose et al. 1975; Westbrook and Sparks 1986; Mitchell et al. 1991). The result was a low-resolution description of migration not much different than that originally suggested by Luginbill (1928), with no practical strategy available for direct testing.

Recently, we described a genetic method that can be used to compare the movements of the overwintering populations from Texas and Florida of the fall armyworm subpopulation (designated the corn-strain [CS]) that primarily infests corn and cotton (Nagoshi et al. 2008). This method uses DNA sequencing information from a portion of the mitochondrial *Cytochrome Oxidase I* (*COI*) gene that is frequently used for DNA barcoding analysis (Hebert et al. 2003). The fall armyworm CS population can be subdivided into four *COI* haplotype classes (CS-h1–CS-h4) as defined by single-base polymorphisms at two sites (Nagoshi et al. 2007). While all four haplotype classes are present in Texas and Florida, there are reproducible differences in the relative proportions of the CS-h4 to CS-h2 frequencies (the h4/h2 ratio). Analysis of Florida populations over a four-year period and over locations spanning the state found an average h4/h2 ratio above 1.5, while the same ratio observed in Texas was consistently less than 0.5 (Nagoshi et al. 2007, 2008). This suggests the existence of reproductive barriers that prevent the homo-genization of the haplotype ratios over time.

Because the polymorphisms that define these haplotypes do not alter the amino acid sequence, significant differences in fitness or behavior are unlikely. Therefore, the ratios characteristic of Florida and Texas should be maintained during migration, thereby acting as a marker of overwintering origin. This was the underlying assumption of test-of-concept surveys examining the distribution of the h4/h2 ratio in the southeastern United States (Nagoshi et al. 2008) and in the northeastern state of Pennsylvania (Nagoshi et al. 2009). These studies described a haplotype ratio pattern suggestive of fall armyworm populations overwintering in Texas being responsible for infestations in most of the United States, west of the Appalachian Mountain range, with Florida-derived populations largely limited to the eastern coastal region. The consistency of these results justified a long-term effort to apply the haplotype ratio method on a larger geographical scale.

Here, we present a summary of fall armyworm collections over a multi-year period (2004–2011) from major corn-producing regions in the southern, central, and eastern United States. The data were used to confirm the ubiquity and consistency of the haplotype frequency profiles derived originally from surveys mostly limited to overwintering populations (Nagoshi et al. 2008). The geographical distribution of the calculated haplotype ratios was used to extrapolate the migratory patterns of the Texas and Florida over-wintering populations and identify areas of overlap and potential hybridization. The migratory pathways indicated and the potential of this method for more detailed monitoring of population movements are discussed.

## Materials and Methods

### Specimen collections and sites

Fall armyworm collections were obtained from either larval collections or pheromone traps using modifications of techniques previously described (Meagher 2001). Some collections were from earlier studies to which more recent sampling data were added (Table 1). Pheromone trapping was performed using standard plastic Universal moth traps (Unitraps) baited with a commercially available fall armyworm pheromone, either a three-component blend specific for fall armyworm (Suterra LLC, Bend, OR) or a two-component mix designated “Fall Armyworm-PSU” lure (Scentry Biologicals, Inc., Billings, MT) that reduces nontarget captures in the northeastern states (Fleischer et al. 2005). Insecticide strips were included to kill specimens (Hercon Environmental Co., Emigsville, PA). After collection, specimens were identified as fall armyworm by morphological criteria and stored at –20°C.

**Table 1:** Source locality and collection information

Location	County	Year	Collector or citation
Alabama (AL) north	DeKalb	2007	R. L. Meagher
AL central	Pooled^1^	2005	Nagoshi et al. (2008)
AL central	Macon	2006–2007	R. L. Meagher
AL south	Coffee	2007	R. L. Meagher
Arkansas (AR)	Pooled	2006	I. Ali, R. Luttrell
Florida (FL) north	Alachua, Levy	2004–2008	Nagoshi et al. (2010)
FL south	Miami-Dade	2003–2007	Nagoshi et al. (2010)
Georgia (GA) north	Newton	2007	R. L. Meagher
GA central	Pike	2004–2006	Nagoshi et al. (2008)
GA central	Peach	2007	R. L. Meagher
GA south	Lowndes	2007	R. L. Meagher
Iowa (IA)	Story	2008–2009	T. Sappington
Illinois (IL)	Champaign, Madison	2008	R. Bellm, K. Steffey
Indiana (IN)	Tippecanoe	2008	J. Obemeyer
Kansas (KS)	Finney	2008	A. Joshi
Kentucky (KY)	Caldwell	2008	D. Johnson, P. Lucas
Louisiana (LA)	Franklin Parish	2007	Nagoshi et al. (2008)
LA	Franklin Parish	2009	B. R. Leonard
Maryland (MD)	Pooled^2^	2008	K. Rice
Mexico	Rio Bravo, Tamaulipas	2007	J. S. Armstrong
Mexico	Torreon, Coahuila	2008	R. Jackson
Minnesota (MN)	Dakota	2011	E. Burkness
Mississippi (MS)	Oktibbeha	2007–2008	R. Jackson
MS	Washington	2004–2005	Nagoshi et al. (2008)
North Carolina (NC)	Henderson	2008	J. Walgenbach
Nebraska (NE)	Clay	2011	R. Wright
New Jersey (NJ)	Pooled^3^	2008	J. Ingerson-Mahar
New York (NY)	Suffolk	2011	D. Gilrein
Oklahoma (OK)	Haskell	2006	T. Royer
Pennsylvania (PA)	Centre	2001, 2006–2007	Nagoshi et al. (2009)
PA	Montgomery	2010	C. Tipping
South Carolina (SC)	Charleston	2011	A. Simmons
Tennessee (TN)	Knox, Robertson	2008	W. Klingeman, F. Hale
TN	Madison	2011	S. Stewart
Texas (TX) central	Brazos	2004	Nagoshi et al. (2008)
TX central	Tom Green	2006	Nagoshi et al. (2008)
TX south	Hidalgo	2006–2008	J. S. Armstrong
TX south	Nueces	2011	R. Parker
Virginia (VA)	Accomack	2008	T. Kuhar

1 Macon, Tallapoosa, Lee.

2 Caroline, Dorchester, Queen Annes, Somerset, Worcester.

3 Cape May, Camden, Warren.

### DNA preparation

Individual specimens were homogenized in 2 mL of phosphate buffered saline (PBS, 20 mM sodium phosphate, 150 mM NaCl, pH 8.0) in a 4-mL test tube using a tissue homogenizer (PRO Scientific Inc., Oxford, CT). Cells and tissue were pelleted by centrifugation at 12,000 *g* for 10 min at room temperature. The pellet was suspended in 500 µL cell lysis buffer (0.2 M sucrose, 0.1 M Tris-HCl at pH 8.0, 0.05 M EDTA, and 0.5% sodium dodecyl sulfate), transferred to a 1.5- or 2.0-mL microcentrifuge tube and incubated at 55°C for 10 min. Proteins were precipitated by the addition of 100 µL of 8 M potassium acetate. The supernatant was transferred to a Zymo-Spin II DNA purification column (Zymo Research, Orange, CA) and processed according to manufacturer's instructions. The DNA preparation was increased to a final volume of 40 µL with distilled water. Each PCR reaction required 1–2 µL of the DNA preparation (approximately 0.02 µg). Genomic DNA preparations of fall armyworm samples from previous studies were stored at –20°C and analyzed as needed (Table 1).

### PCR amplification

PCR amplification of the *COI* gene was performed in a 30 µL reaction mix containing 3 µL 10× manufacturer's reaction buffer, 0.5 µL 10 mM dNTP, 0.5 µL 20 µM primer mix, 1–2 µL DNA template (between 0.05 and 0.5 µg), 0.5 unit Taq DNA polymerase (New England Biolabs, Beverly, MA). The thermocycling program was 94°C (1 min), followed by 33 cycles of 92°C (30 sec), 56°C (45 sec), 72°C (45 sec), and a final segment of 72°C for 3 min. Typically 96 PCR amplifications were performed at the same time using either 0.2-mL tube strips or 96-well microtiter plates. Amplification of the *COI* region used the primer pair *COI-893F* (5′-CACGAGCATATTTTACATCWGCA-3′) and *COI-1303R* (5′-CAGGATAGTCAGAATATCGACG-3′) to produce a 410-bp fragment (Integrated DNA Technologies, Coralville, IA).

### Strain identification and DNA sequence analysis

**Figure 2:**
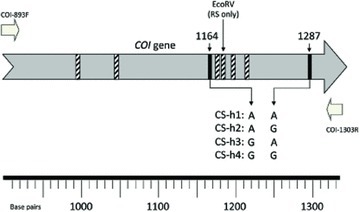
Diagram of the portion of mitochondrial *Cytochrome Oxidase I* (*COI*) gene used to identify strain and the individual corn-strain (CS) haplotypes. The putative translational start site of the *COI* gene was arbitrarily designated as coordinate 0. Short block arrows indicate location and direction of the COI-893F and COI-1303R primers used for PCR amplification and DNA sequencing. Vertical lines within the *COI* gene identify polymorphic sites with the rice-strain (RS) specific EcoRV site identified. All polymorphic sites except 1287 were used to identify or confirm strain identity. The CS-h haplotypes were determined by the polymorphisms at site 1164 and 1287.

In each PCR, reaction mix (30 µL) was added 5 units of the restriction enzyme EcoRV (New England Biolabs) and 4 µL of the manufacturer recommended 10× restriction enzyme buffer (final volume taken to 40 µL with water). Restriction digests were incubated at 37°C 1–3 h. For each reaction, 6 µL of 6× gel loading buffer was added and the entire sample run on a 1.8% agarose horizontal gel containing GelRed per manufacturer's instructions (Biotium, Hayward, CA) in 0.5× Tris-borate buffer (TBE, 45 mM Tris base, 45 mM boric acid, 1 mM EDTA pH 8.0). Fragments were visualized on a long-wave ultraviolet light box. Only the rice-strain (RS) associated *COI* allele has an EcoRV in the amplified region (Fig. 2). Therefore, uncut fragments were of the CS and these were isolated using the Zymoclean Gel DNA Recovery kit (Zymo Research). Strain identification was confirmed by DNA sequence analysis (Northwoods DNA, Inc., Bemidji, MN; and the University of Florida ICBR center).

### Analyzing haplotypes

DNA sequence information at sites 1164 and sites 1287 in the *COI* region were used to identify the CS-h1–4 haplotypes (Fig. 2). In the CS, each site was empirically found to be associated with two alternative bases to produce four possible haplotypes: CS-h1 (A_1164_A_1287_), CS-h2 (A_1164_G_1287_), CS-h3 (G_1164_A_1287_), and CS-h4 (G_1164_G_1287_). The frequencies of the four haplotypes were calculated for each collection, as was the quotient of the CS-h4 to CS-h2 frequencies (designated the h4/h2 ratio). We have found that 15 specimens generated a frequency profile that does not substantially change with additional sampling. DNA comparisons, alignments, and restriction site mapping were performed using the DS Gene program (Accelrys, San Diego, CA) and the Geneious Pro 5.4.6. Program (Drummond et al. 2010). Statistical analyses were performed using GraphPad InStat version 5.1 (GraphPad Software, San Diego, CA, http://www.graphpad.com). Pairwise comparisons were performed using the nonparametric, two-tailed, Mann–Whitney *U* test. The nonparametric *t*-test was used because several datasets failed the normality test. Correlation tests were performed using Pearson's correlation.

## Results

### Consistency of haplotype frequency relationships

Collections from over 30 locations in major corn-producing areas of the United States were assessed for their CS-h1–4 haplotype profiles (Table 2). On average, almost 90% of the fall armyworm CS population was made up of CS-h2 (53%) and CS-h4 (36%), with each haplotype displaying substantial variability. The CS-h2 frequencies ranged from 9% to 88% and CS-h4 from 5% to 85%. Pairwise distributions of the CS-h2 and CS-h4 haplotypes showed a highly significant negative correlation (Pearson *r* = –0.98; *P* < 0.0001). The CS-h1 and CS-h3 haplotypes were generally infrequent, averaging 10% and 1% of the population, respectively (Table 2). Similar haplotype proportions have been observed in surveys of fall armyworm populations from Puerto Rico to South America (Nagoshi et al. 2007, 2010, 2012).

**Table 2:** Haplotype frequencies and the haplotype ratios for different locations

				CS haplotype frequency						
							
Overwintering group^1^	State/region	Year	Total	h1	h2	h3	h4	h4/h2	[G/A]_1164_	%Δ^2^
TX-group	TX[M]^3^									
(h4/h2 <0.6)	AL central	2005–2007	299	0.11	0.59	0.01	0.30	0.11	0.4	−14
	AL north	2006	68	0.10	0.60	0.04	0.25	0.10	0.4	0
	IA	2008–2009	90	0.16	0.71	0.00	0.13	0.16	0.2	−18
	IL	2008	63	0.11	0.68	0.00	0.21	0.11	0.3	−14
	IN	2008, 2011	121	0.13	0.69	0.00	0.17	0.13	0.2	−16
	KS	2008	32	0.19	0.72	0.00	0.09	0.19	0.1	−21
	KY	2008	29	0.10	0.69	0.00	0.21	0.10	0.3	−13
	LA	2007, 2009	42	0.05	0.88	0.00	0.07	0.05	0.1	−5
	MN	2011	27	0.07	0.70	0.00	0.22	0.07	0.3	−10
	MS	2004–2005	205	0.12	0.63	0.00	0.24	0.12	0.3	−16
	MS	2007–2008	62	0.21	0.50	0.02	0.27	0.21	0.4	−25
	NE	2011	83	0.11	0.72	0.00	0.17	0.11	0.2	−13
	OK	2006	68	0.18	0.69	0.00	0.13	0.18	0.2	−20
	PA	2001, 2006–2007	278	0.17	0.67	0.00	0.16	0.17	0.2	−20
	PA	2010	26	0.19	0.58	0.00	0.23	0.19	0.3	−25
	TN	2008	41	0.07	0.63	0.02	0.27	0.07	0.4	−2
	TX central	2004	141	0.16	0.72	0.00	0.12	0.16	0.1	−19
	TX central	2006–2007	41	0.15	0.68	0.00	0.17	0.15	0.2	−18
	TX[O]^3^									
	TX south	2006	58	0.10	0.66	0.03	0.21	0.3	0.3	1
	TX south	2007	62	0.10	0.82	0.00	0.08	0.1	0.1	−11
	TX south	2008	40	0.10	0.68	0.00	0.23	0.3	0.3	−13
	TX south	2011	63	0.08	0.65	0.00	0.27	0.4	0.4	−11
	Mexico	2006–2007	72	0.14	0.78	0.00	0.08	0.1	0.1	−15
	Mexico	2008	21	0.00	1.00	0.00	0.00	0.0	0.0	0
Intermediate	NJ	2008	156	0.04	0.54	0.01	0.41	0.8	0.7	−6
(h4/h2 = 0.6–1.3)	NY	2011	44	0.11	0.43	0.00	0.45	1.1	0.8	−21
	GA north	2007	97	0.13	0.48	0.00	0.38	0.8	0.6	−22
	AL south	2007	189	0.05	0.48	0.01	0.47	1.0	0.9	−7
FL-group	FL[M]^3^									
(h4/h2 >1.3)	FL north	2005	98	0.03	0.27	0.02	0.68	2.6	2.4	−8
	FL north	2006	60	0.07	0.27	0.00	0.67	2.5	2.0	−20
	FL north	2007	32	0.00	0.41	0.00	0.59	1.5	1.5	0
	FL north	2008	31	0.10	0.23	0.00	0.68	3.0	2.1	−30
	FL central	2011	37	0.05	0.14	0.00	0.81	6.0	4.3	−29
	GA central	2007	131	0.14	0.34	0.01	0.52	1.6	1.1	−28
	GA south	2007	127	0.07	0.30	0.02	0.61	2.1	1.7	−17
	MD	2008	21	0.05	0.38	0.05	0.52	1.4	1.3	−3
	SC	2011	71	0.04	0.27	0.00	0.69	2.6	2.2	−14
	NC	2004–2008	157	0.09	0.38	0.01	0.53	1.4	1.2	−18
	VA	2008	73	0.15	0.33	0.03	0.49	1.5	1.1	−28
	FL[O]3									
	FL south	2004	56	0.13	0.09	0.02	0.77	8.6	3.7	−57
	FL south	2005	57	0.11	0.26	0.00	0.63	2.4	1.7	−29
	FL south	2006	65	0.03	0.12	0.00	0.85	6.9	5.5	−20
	FL south	2007	44	0.02	0.25	0.02	0.70	2.8	2.7	−5

1 Overwintering source inferred from the h4/h2 ratio.

2 %Δ, percent change between h4/h2 (A) ratio and [G/A]_1164_ (B) ratio as calculated by (B – A)/A × 100.

3 [M] and [O] refers to migratory and overwintering populations, respectively (see text).

### Nonrandom spatial distribution of haplotype ratios

The h4/h2 frequency ratio was the metric previously developed to distinguish between fall armyworm populations (Nagoshi et al. 2008). A summary of data from the published studies listed in Table 1 showed an average ratio of 0.2 for Texas and 3.8 for Florida for collections made from 2004 to 2008. Surveys of Nueces Co., TX (0.4) and Orange Co., FL (6.0) demonstrated that this regional difference had persisted into 2011 (Table 2). The h4/h2 ratios for all collections listed ranged from 0.0 to 8.6, with an average value of 1.4.

**Figure 3:**
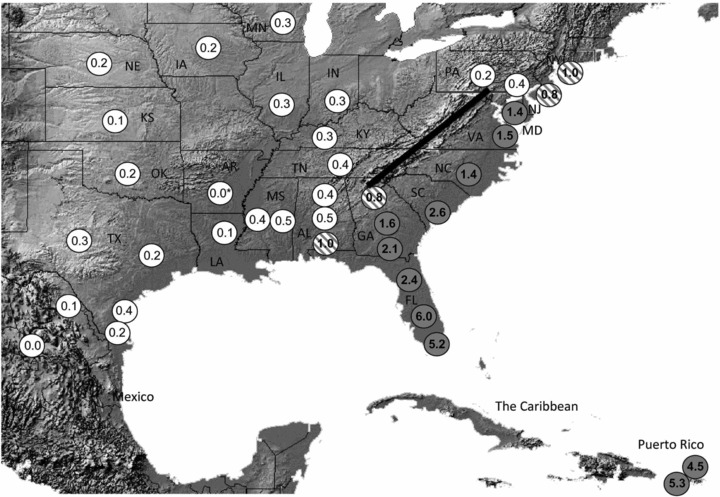
Map summarizing the geographical distribution of h4/h2 haplotype ratios from collections listed in Table 2. Ratios from central and southern Texas and southern Florida representing multiple years from the same locations were averaged. Shaded circles indicate the FL group with ratios >1.0. Open circles depict the TX group defined by ratios <0.6. Circles with diagonal lines identify collections with intermediate ratios. Haplotype ratios for Puerto Rico are from Nagoshi et al. (2010). The asterisk identifies the ratio obtained from 13 specimens collected at several sites in Arkansas. Because of the small sample size, these data were not included in the analyses in Table 2 or 3, but is presented here to show consistency with neighboring areas. Diagonal line follows the major elevations of the Appalachian Mountain range. Map courtesy of the United States Geological Survey.

Mapping of the ratios revealed a nonrandom geo-graphical distribution (Fig. 3). Collections with higher ratios (≥1.4) characteristic of Florida overwintering populations were clustered along the Atlantic coast from Florida to Maryland, and included parts of Georgia, South Carolina, North Carolina, and Virginia. These had an average ratio of 3.1 and were designated the FL group (Table 2). In contrast, all sites west of the Appalachian Mountain range had ratios ≤0.5, with an average of 0.3 that is diagnostic of fall armyworm that overwinter in southern Texas and Mexico. These collections were categorized as the TX group. Ratios outside these two categories (0.5 > ratio < 1.4) were found in four collections located near the north central (NJ, NY) or southern (GA north, AL south) portions of the Appalachian Mountain range (Fig. 3). These were grouped separately and designated as “Intermediate” (Table 2). All three groups showed highly significant (*P* < 0.001) differences in pairwise comparisons of their haplotype ratios as well as in the individual CS-h2 and CS-h4 frequencies (Table 3).

**Table 3:** Statistical comparisons between the average haplotype ratios and frequencies from data listed in Table 2. Standard deviations are in parentheses

	Average frequency					
	
Group	h4/h2	CS-h1	CS-h2	CS-h3	CS-h4	[G/A]_1164_
Total	1.4 (1.9)	0.10 (0.05)	0.53 (0.22)	0.01 (0.01)	0.36 (0.24)	1.0 (1.2)
TX group (TX)	0.3 (0.1)	0.12 (0.05)	0.69 (0.10)	0.01 (0.01)	0.18 (0.08)	0.3 (0.1)
FL group (FL)	3.1 (2.2)	0.07 (0.05)	0.27 (0.10)	0.01 (0.01)	0.65 (0.11)	2.3 (1.3)
Intermediate (Int)	0.9 (0.1)	0.08 (0.04)	0.48 (0.05)	0.00 (0.01)	0.43 (0.04)	0.8 (0.1)
TX[O]	0.2 (0.2)	0.09 (0.05)	0.77 (0.13)	0.01 (0.01)	0.15 (0.11)	0.2 (0.2)
TX[M]	0.3 (0.1)	0.13 (0.05)	0.67 (0.08)	0.01 (0.01)	0.19 (0.07)	0.3 (0.1)
FL[O]	5.2 (3.1)	0.07 (0.06)	0.18 (0.09)	0.01 (0.01)	0.74 (0.09)	3.4 (1.6)
FL[M]	2.4 (1.3)	0.07 (0.05)	0.30 (0.08)	0.01 (0.02)	0.62 (0.10)	1.9 (0.9)

	Two-tailed *P*-value					
	
Comparisons	h4/h2	CS-h1	CS-h2	CS-h3	CS-h4	[G/A]_1164_
TX versus FL	*P* < 0.0001	*P* = 0.006	*P* < 0.0001	*P* = 0.11	*P* < 0.0001	*P* < 0.0001
TX versus Int	*P* = 0.0001	*P* = 0.18	*P* = 0.0002	*P* = 0.56	*P* = 0.0001	*P* = 0.0001
FL versus Int	*P* = 0.0005	*P* = 0.69	*P* = 0.0005	*P* = 0.51	*P* = 0.0005	*P* = 0.0005
TX[O] versus TX[M]	*P* = 0.37	*P* = 0.06	*P* = 0.24	*P* = 0.92	*P* = 0.39	*P* = 0.40
FL[O] versus FL[M]	*P* = 0.06	*P* = 0.90	*P* = 0.02	*P* = 0.95	*P* = 0.06	*P* = 0.06

### Comparison of overwintering and migratory haplotype profiles

The overwintering range of fall armyworm in the United States is limited to southern Texas and southern Florida during normal winters (Luginbill 1928). The northern limit for the Florida overwintering zone was previously suggested to be 28°N latitude (Pair and Sparks 1986), which in this study was represented by trapping sites in Miami-Dade county (designated FL[O]). Application of the same latitude limit to Texas placed the sites in Hidalgo and Nueces counties within the Texas overwintering region (TX[O]). The average haplotype ratio and frequencies were compared for FL[O] and TX[O] with those at the respective migratory (FL[M], TX[M]) destinations (Table 2). No significant differences were observed between TX[O] and TX[M] (Table 3). The analysis of the FL group was less conclusive. There was a substantial difference in the average haplotype ratio at the Florida overwintering locations (5.17) compared to the FL[M] group (2.36), although we note that the former was associated with large annual fluctuations ranging from a low of 2.40 to a high of 8.60 (Table 2). This ratio difference approached significance (*P* = 0.06), as did the comparison of CS-h4 frequencies, but statistical significance was only observed for CS-h2 (*P* = 0.02).

### Simplified ratios to study migration

The adoption of the h4/h2 metric was based on our initial observations that CS-h1 frequencies were too variable to consistently identify regional differences and that CS-h3 was too rare to be of relevance (Nagoshi et al. 2008). However, using the larger database developed in this study, a significant difference (*P* = 0.006) in the frequency of CS-h1 was observed between the TX group and FL group (Table 3). Furthermore, the CS-h1 profile showed a significant positive correlation (*r* = 0.35, *P* = 0.03) with CS-h2, indicating they shared the same regional biases. These results suggest that it may not be necessary to distinguish between CS-h1 and CS-h2, which differ only at site 1287 (Fig. 2). The haplotype procedure could then be simplified by a new metric that only compares the G and A frequencies at site 1164 (i.e., the [G/A]_1164_ ratio), effectively pooling CS-h1 with CS-h2. This would also pool CS-h3 with CS-h4, but the former is so infrequent as to be inconsequential. To assess the effectiveness of this modification, [G/A]_1164_ was calculated for all collections (Table 2). The average difference between the h4/h2 and [G/A]_1164_ ratios was –16% with a change as high as –57% observed (Table 2). Nevertheless, the [G/A]_1164_ ratio showed a very strong positive correlation with the h4/h2 profile (*r* = 0.95, *P* < 0.0001) and did not alter the compositions of the intermediate group, the TX group or the FL group (Table 2). Statistical comparisons between groups using [G/A]_1164_ were comparable for all analyses (Table 3). These results indicate that the two metrics are equivalent at distinguishing between regional populations, with [G/A]_1164_ having the advantage of requiring less DNA sequence information.

## Discussion

These studies demonstrate that mitochondrial haplotypes ubiquitous to all populations examined can still be developed as region-specific genetic markers if there are consistent differences in their relative frequencies. Such variation can occur for many reasons, including but not limited to genetic drift and population bottlenecks, and once formed can persist indefinitely if mixing between groups is sufficiently limited. This appears to be the case for fall armyworm despite their capacity for long-distance flight. Our results indicate that the frequency differences observed between Texas and Florida populations beginning in 2004 collections have persisted into 2011.

### Fall armyworm migratory pathways

**Figure 4:**
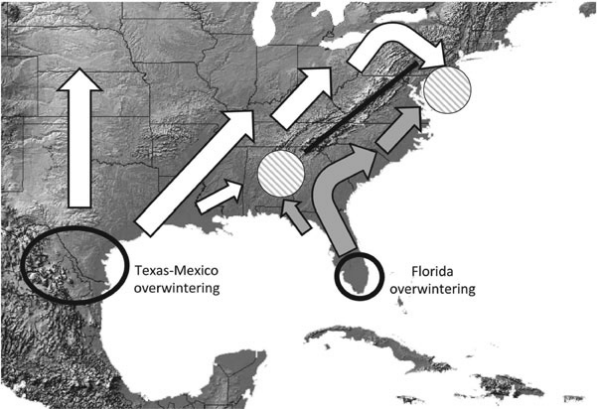
Same map as in Figure 2 showing migratory pathways inferred from the geographical distribution of haplotype ratios. Open ovals estimate the extent of the overwintering range in Texas and Florida. Fall armyworm can overwinter in the Caribbean but whether these populations contribute to those in Florida is a matter of speculation (see text). White arrows indicated putative direction of the migration from the Texas–Mexico overwintering areas. Shaded arrows depict movement from Florida populations. Lined circles approximate locations of “hybrid zones” where the two migratory pathways appear to overlap. Diagonal line follows the major elevations of the Appalachian Mountain range.

The spatial distribution of the haplotype ratios was highly asymmetric and compartmental. A pattern of low (≤0.5) ratios was observed throughout Texas and the central United States, extending eastward to the Appalachian Mountain range, while haplotype ratios ≥1.4 defined a contiguous region comprising Florida, Georgia, South Carolina, North Carolina, Virginia, and Maryland (Fig. 3). The similarity of these regional ratio averages to that characteristic of Texas and Florida overwintering populations are consistent with discrete and progressive migratory pathways originating from the overwintering locations (summarized in Fig. 4). Texas populations migrate northward into the central states of Kansas, Nebraska, and Iowa and to the northeast following the Mississippi River Valley into Illinois and Minnesota, extending eastward to Pennsylvania. In contrast, migration from Florida is limited to the more southern Atlantic coastal states, with no evidence of substantial contributions to infestations occurring west of the Appalachian Range.

That this study was able to delineate such well-defined haplotype distribution patterns strongly suggest consistent year-to-year migration pathways. For practical reasons, the data mapped in Figure 3 were from collections made in different years, ranging from 2004 to 2011, and in some cases represented averages of collections made at different times of the year or over multiple years. Therefore, regional asymmetries in haplotype frequencies would have been obscured if significant seasonal or yearly variations in the migratory pathways were common.

### Regions of migratory overlap

Areas of overlap between the Texas and Florida migratory waves are of potential biological importance as they represent the primary opportunity for genetic exchange between the two overwintering regions. Based on haplotype ratios, the observed hybrid zones are relatively discrete. In the northeast, intermediate ratios were observed in New Jersey and eastern New York, while the adjacent states of Pennsylvania and Maryland had the TX group and FL group ratios, respectively (Fig. 3). In the south, the hybrid region appears to be localized to the Alabama–Georgia region. These observations suggest the existence of migratory boundaries that are relatively well defined, at least at the state level of resolution. This separation may be in large part due to the Appalachians acting as a physical barrier both in terms of elevation and as a region devoid of large corn acreages, the preferred host plant of the fall armyworm corn strain. Phylogeographical discontinuities across the Appalachian Mountains have been observed for a number of other taxa, although these cases represent boundaries in the migration and establishment of permanent populations (reviewed in Soltis et al. 2006). Whether similar factors are involved in the recurring segregation of fall armyworm migrations remains to be elucidated.

### Introgression of Texas fall armyworm into Florida

Comparisons of the haplotype frequencies in the southern Florida overwintering region (FL[O]) with that of the migratory (FL[M]) destinations showed a significant (*P* = 0.02) increase in the frequency of CS-h2, the haplotype that is most common in the TX group. This suggests substantial mixing of Texas migrants with those from Florida in the hybrid zones to produce intermediate haplotype ratios. The long-term consequences of such interactions depends on the degree to which the Texas migrants and hybrids enter the Florida overwintering sites, as only the overwintering population contributes genetically to the next generation. We have frequently observed a gradient of haplotype ratios, with higher values in southern Florida than in northern Florida, suggesting a southward progression of Texas haplotypes (Fig. 3; Nagoshi et al. 2010). In contrast, there is no evidence of a reciprocal introgression of Florida haplotypes into Texas, as TX[O] and TX[M] haplotype frequencies are statistically identical (Table 3).

Repeated introgression of Texas migrants into the Florida overwintering population should eventually result in the homogenization of the haplotype ratios between the two states. That this is not occurring suggests either that the Texas contribution is insignificant, or that the Florida haplotype profile is being maintained by regular additions from an external source. A likely candidate for the latter possibility is the Caribbean, as high h4/h2 ratios were consistently observed in Puerto Rico fall armyworms (Nagoshi et al. 2010). While atmospheric trajectories favorable for the northward transport from the Caribbean to the southeastern United States have been reported (Westbrook and Sparks 1986), a detailed study of trap captures associated with synoptic wind patterns consistent with such movements was not conclusive (Mitchell et al. 1991). More detailed comparisons of the haplotype frequencies in Florida and the Caribbean could be informative.

In summary, the haplotype ratio method consistently detects significant differences in haplotype frequencies in the fall armyworm CS population that overwinter in southern Texas and Mexico from those observed in southern Florida and Puerto Rico. These differences are maintained in the migratory populations, allowing for the first time a detailed comparison of the movements from the two major over-wintering regions across the central and eastern United States. The migration of the Florida fall armyworm is restricted to regions east of the Appalachian Mountain range. Overlap between these populations and those originating from Texas, appears to be limited to relatively small areas north and south of the primary elevations of the Appalachians. This suggests limited opportunities for genetic exchange, consistent with the observed stability of the haplotype frequency asymmetries over time. These findings have important implications for estimating the potential threat of invasive populations carrying deleterious alleles that might become established in southern Florida.
